# *Candida* species and selected behavioral factors co-associated with severe early childhood caries: Case-control study

**DOI:** 10.3389/fcimb.2022.943480

**Published:** 2022-07-25

**Authors:** Michaela Cvanova, Filip Ruzicka, Martina Kukletova, Bretislav Lipovy, Daniela Gachova, Lydie Izakovicova Holla, Zdenek Danek, Veronika Hola, Michaela Bartosova, Jiri Jarkovsky, Ladislav Dusek, Petra Borilova Linhartova

**Affiliations:** ^1^ RECETOX, Faculty of Science, Masaryk University Kotlarska 2, Brno, Czechia; ^2^ Institute of Biostatistics and Analyses, Faculty of Medicine, Masaryk University, Brno, Czechia; ^3^ Clinic of Microbiology, Institution Shared with St. Anne´s University Hospital, Faculty of Medicine, Masaryk University, Brno, Czechia; ^4^ Clinic of Stomatology, Institution Shared with St. Anne´s University Hospital, Faculty of Medicine, Masaryk University, Brno, Czechia; ^5^ Clinic of Burns and Plastic Surgery, Institution shared with University Hospital Brno, Faculty of Medicine, Masaryk University, Brno, Czechia; ^6^ Clinic of Maxillofacial Surgery, Institution shared with the University Hospital Brno, Faculty of Medicine, Masaryk University, Brno, Czechia; ^7^ Department of Pathophysiology, Faculty of Medicine, Masaryk University, Brno, Czechia

**Keywords:** severe early childhood caries (sECC), *Candida* sp., *Streptococcus mutans*, breastfeeding, sweet beverages, brushing of teeth, *Candida dubliniensis*

## Abstract

Severe Early Childhood Caries (sECC) is a multifactorial disease associated with the occurrence of specific oral microorganisms and other environmental, behavioral, and genetic factors. This study aimed to construct a multivariable model including the occurrence of *Candida* spp. and selected behavioral factors (length of breastfeeding, serving sweet beverages and beginning of brushing child’s teeth) to determine their relationships to the occurrence of sECC.

In this case-control study 164 children with sECC and 147 children without dental caries were included. MALDI-TOF MS and multiplex qPCR were used to identify *Candida* spp. and selected bacteria in dental plaque samples, respectively. A questionnaire on oral hygiene, diet, and children’s health was filled in by the parents.

The constructed multivariable logistic regression model showed an independent influence of the microbial and behavioral factors in sECC etiopathogenesis. The occurrence of *C. albicans* and *C. dubliniensis* was associated with higher odds of sECC development (odds ratio, OR: 9.62 and 16.93, respectively), together with breastfeeding of 6 months or less (OR: 2.71), exposure to sweet beverages (OR: 3.77), and starting to brush child’s teeth after the 12^th^ month of age (OR: 4.10), all statistically significant (*p* < 0.01).

Considering the high occurrence of *C. albicans* and *C. dubliniensis* in dental plaque in children with sECC, we propose them as “keystone pathogens” and risk factors for sECC. The models showed that presence of specific species of *Candida* in dental plaque may be a better descriptor of sECC than the mentioned behavioral factors.

## 1 Introduction

Untreated dental caries in permanent teeth is the worldwide most prevalent chronic disease, while Early Childhood Caries (ECC) was the 10^th^ most prevalent condition, affecting 9% of the global child population between 1990 and 2010 and 7.8% in 2015 according to the Global Burden of Diseases Studies (Kassebaum et al., 2017). Caries in the primary dentition is referred to as ECC, its severe form (sECC) is defined using a combination of the age of the child and the number of teeth that are cavitated/missing due to caries/filled. In a 5-year-old child, sECC is diagnosed if 6 or more teeth in the primary dentition are affected by dental caries (AAPD, 2017). The composition of the oral microbiome is unique for each individual and reflects a variety of endogenous and exogenous influences (genetic, immunological, behavioral, and environmental factors) (O’Donnell et al., 2015).

The oral environment is a complex dynamic system in which any deviation can through compensatory mechanisms lead to the development of a wide range of potentially pathological conditions. Complex host–diet–microbe interactions initiate the formation of a virulent biofilm on the teeth and may lead to the emergence of cavitation or carious lesions (Hajishengallis et al., 2017). Hajishengallis et al. (2017) suggested that microbiome and salivary biomolecule analyses could be combined with behavioral risk assessments to improve the accuracy of the existing ECC-risk screening methods and provide greater predictability of ECC development. In a meta-analysis by Kirthiga et al. (2019), 123 risk factors of ECC were found; high levels of the facultatively anaerobic Gram-positive bacterium *Streptococcus mutans*, visible dental plaque, poor oral hygiene, and frequent consumption of sweetened foods were among the main factors associated with the onset and development of ECC. It is now generally accepted that synergistic interactions between oral streptococci and *Candida albicans* (or non-albicans candida species) play a pivotal role in the etiopathogenesis of ECC development (Bachtiar and Bachtiar, 2018; Chevalier et al., 2018).

*C. albicans* is one of the most commonly detected fungi in human mucous membranes; in the oral cavity, this yeast is known to colonize both viable and prosthetic surfaces and to cause oral mucosal infections (Samaranayake et al., 2009; Lalla et al., 2013). *Candida dubliniensis*, one of the non-albicans *Candida*, was first isolated from the oral cavity of HIV-positive patients in Dublin (Ireland) in 1995 (Sullivan et al., 1995). *C. dubliniensis* most commonly colonizes the oral cavity and upper respiratory tract (Moran et al., 2012; Al-Ahmad et al., 2016). In the context of dental caries, *C. dubliniensis* was first isolated from the plaque and carious dentine of a healthy 5-year-old boy (Kneist et al., 2015).

The characteristics of *C. dubliniensis* are very similar to those of *C. albicans*, including the formation of germ tubes and chlamydospores (Stokes et al., 2007; Ells et al., 2011). The representation of individual candidas in the oral cavity is highly disputable as they are difficult to distinguish using standard microbial methods. The closeness in one of the principal factors of the virulence of both species, i.e., their dimorphism (ability to switch between the yeast and filamentous growth forms), led to the fact that they have been for a long time considered a single species (Baillie and Douglas, 1999; Jabra-Rizk et al., 2006; Ayadi et al., 2020). One significant difference between *C. albicans* and *C. dubliniensis* is the inability of the latter to express β-glucosidase activity (Schoofs et al., 1997; Sullivan et al., 1997). Routine introduction of MALDI-TOF (Matrix-Assisted Laser Desorption/Ionization, Time-Of-Flight) into the clinical practice has greatly improved the accuracy of the diagnosis of both these candida species (Hof et al., 2012) and, hence, facilitated the studies of the separate associations of these species with dental caries. However, studies distinguishing between *C. albicans* and *C. dubliniensis* in this respect are still relatively rare and their results are inconsistent.

In two meta-analyses, *Candida* spp., especially *C. albicans*, were associated with ECC as well as with dental caries in permanent dentition (Xiao et al., 2018b; Eidt et al., 2020). Moreover, in the recent studies by Al-Ahmad et al. (2016) and de Jesus et al. (2020), *C. dubliniensis* was found to be significantly more abundant in the dental plaque of children with ECC, especially those with sECC, compared to caries-free children. In the mycobiome study by O’Connell et al. (O’Connell et al., 2020), the abundance of this emerging opportunistic pathogen increased steadily with the degree of caries progression, which led the authors to suggest that *C. dubliniensis* may play an important role in dental caries pathogenicity. We hypothesize that individual *Candida* spp. support and stabilize the microbiota associated with disease, and thus may be referred to as “keystone pathogens” for sECC (Hajishengallis et al., 2012).

Whereas most previous works focused on the occurrence of *Candida* sp. regardless of the speciation (or used methods unsuitable for differentiation between *C. albicans* and *C. dubliniensis*), we decided in this study (i) to investigate individual *Candida* spp. in the dental plaque of preschool children by using a method capable of distinguishing *C. albicans* from *C. dubliniensis*. (ii) We aimed to compare their occurrence in sECC children to that in caries-free children using robust methods as previous studies in smaller cohorts reported inconsistent results. (iii) Finally, no multivariable model for prediction of the risk for sECC that would include colonization by fungi is available at present; thus, we aimed to perform a complex evaluation of both microbial (bacterial and mycobial) and behavioral risk factors for sECC and to propose such a multivariable model. To maximize the accuracy of the results, we applied strict exclusion criteria for patients’ enrolment and compared only two extreme phenotypes.

## 2 Materials and methods

### 2.1 Studied subjects, inclusion criteria, clinical examination and sample collection

All patients attending the participating dental clinics in South Moravia, Czech Republic, in 2005–2020 who met the inclusion criteria were offered participation. All such patients whose parents or legal guardians agreed and signed informed consent were included in the study (N = 632 children) and 487 of them underwent fungal analysis. The study was approved by the Ethics Committees of the Masaryk University (No. 3/2004, 3^rd^ March 2004) and of the St. Anne’s University Hospital in Brno, Czech Republic (No. 1G/2017, 24^th^ June 2016).

Oral clinical examination was performed by dentists. A radiographic examination was not performed as it was not a part of the routine dental care for these subjects and would therefore be deemed unethical. Children who were unable to undergo standard dental treatment due to their uncooperativeness and the need for multiple restorations and/or extractions were examined and treated under general anesthesia. The dmft index (number of decayed, missing, and filled teeth) was calculated using dental caries (d_3_ level) as a cut-off point for the detection of decay (Borilova Linhartova et al., 2016; Borilova Linhartova et al., 2018).

This study was designed as a case-control association study. Children were classified into two groups according to the status of their primary dentition: a group of controls (children with dmft = 0 older than 2 years) and a group of cases (children with dmft ≥ 6, i.e., children with sECC according to AAPD, 2017). As many controls as cases were enrolled in the study to ensure the balanced numbers of participants in the groups. To be better able to evaluate the association between the factors and dental caries, we decided to include only the two extreme groups in this case-control study. For this reason, children with low caries experience in the primary dentition (1 ≤ dmft ≤ 5) were excluded from the study.

In **Supplementary Figure S1**, an overall diagram describing children’s recruitment and selection is presented. The inclusion criteria for participants were preschool age (up to 6 years), general good health, and the willingness of the parents or legal guardians to enter their children into the study. The following exclusion criteria were applied: the presence of one or more permanent teeth, number of unerupted teeth > 4 and/or the number of missing teeth ≥ 10, a close familial relationship between children, and ethnicity other than Czech Caucasian to maintain genetic homogeneity. Additional exclusion criteria covered aspects affecting the immune system, which could affect the oral microbiome, namely: birth weight < 2500 g, oncological disease, diseases associated with immune system impairment (specifically children with asthma, leucinosis, rheumatoid arthritis, PFAPA (periodic fever, aphthous stomatitis, pharyngitis, adenitis) syndrome, idiopathic bowel disease, and inflammation of kidney), any congenital developmental disorder (cleft palate and/or lip, etc.), severely underweight children with z-score < -3 and severely obese children with z-score > 3 according to the WHO child growth standards (The WHO, 2006). Children who used antimicrobials in the last three months before examination and sample collection were also excluded from the study.

The parent or legal guardian filled in a questionnaire relating to the oral hygiene of the child and its eating/drinking habits (age at the beginning of teeth brushing, daily frequency of teeth brushing, age at the time of weaning, nocturnal breastfeeding, and the consumption of sweet beverages). Subjects with missing data in the questionnaire (a question on nocturnal breastfeeding) were removed from the study. Where “eruption of the first tooth” was entered as the time of the beginning of tooth brushing, the mean age at the eruption of the first tooth according to American Dental Association, i.e., 8 months (The ADA, 2005), was assigned to such records for further analyses.

A supragingival dental plaque sample from both the upper and lower teeth for microbial (fungal) analysis was obtained from each patient by a sterile swab and immediately placed in Amies medium (COPAN Italia, Microbiology swab Transystem™). Samples were stored at 4°C and analyzed within 24 hours.

Subgingival and supragingival dental plaque samples were collected from the year 2016 for the microbial (bacterial) analysis. The tooth No. 73 was considered a “control tooth” in the group of controls, one of the teeth affected by dental caries was selected as an “affected tooth” in the group of cases. Sampling was performed using a paper cone (ISO 40); the tooth was encircled by the cone until it was covered with up to 2/3 with the biological material. Each paper cone was separately placed into a sterile tube using tweezers and stored at −20°C.

### 2.2 Microbial analysis

#### 2.2.1 Identification of *Candida* spp.

MALDI-TOF MS was used for the identification of *Candida* spp. Dental plaque samples from swabs were resuspended in 1 mL phosphate buffered saline (PBS) and homogenized by vortexing. Subsequently, 100 μL of the resultant homogenate was inoculated onto CHROMagar Candida (CHROMagar, Paris, France) and incubated for 48 hrs at 37°C. After the incubation the growth of yeast was assessed. All isolates were identified by MALDI-TOF using the extended Direct Transfer method according to the manufacturer’s instructions (Bruker; MALDI Biotyper Protocol Guide; Edition 2, 2014). Single colonies were applied as a thin film onto a target of the MALDI 96-target plate (Bruker Daltonics, Billerica, MA, USA) and overlaid with 1 μL formic acid. The dried sample was overlaid with 1 μL of the matrix solution, a saturated α-cyano-4-hydroxycinnamic acid (Bruker Daltonics, Billerica, MA, USA) solution in a mixture of acetonitrile:water:trifluoroacetic acid (50:47.5:2.5, v/v), and allowed to dry before testing.

MALDI-TOF MS measurements were carried out with a MALDI BioTyper system (Bruker Daltonics) and FlexControl 3.4 software (Bruker Daltonics, Billerica, MA, USA). Mass spectra were processed using BioTyper 3.1 software (Bruker Daltonics, Billerica, MA, USA).

The manufacturer-recommended cut-off scores were used for identification, with scores of ≥ 2.000 indicating identification to the species level, scores between 1.700 and 1.999 indicating identification to the genus level, and scores of < 1.700 indicating no identification. The isolates producing scores of < 1.700 were retested, and the highest score was used for identification.

#### 2.2.2 Molecular analysis of selected oral bacteria

Genomic DNA was isolated from subgingival and supragingival dental plaque samples using QIAamp DNA Mini Kit (Qiagen, Hilden, Germany) according to the manufacturer’s recommendations. The presence of three cariogenic bacteria (*Streptococcus mutans*, *Lactobacillus* sp., and *Actinomycetes* sp.) and 7 periodontal bacteria (*Aggregatibacter actinomycetemcomitans*, *Porphyromonas gingivalis*, *Tanarella forsythia*, *Treponema denticola*, *Parvimonas micra*, *Prevotella intermedia*, and *Fusobacterium nucleatum*) was analyzed in the subsample by a multiplex Real-Time polymerase chain reaction method (see **Supplementary Figure S1**). The method was originally developed and published by our team previousl (Lochman et al., 2019); samples from 30 children from this study were also used in the current study.

### 2.3 Statistical analysis

The data were analyzed in IBM SPSS Statistics for Windows, version 27, unless otherwise stated. Basic characteristics of patients were described using standard descriptive statistics. Where the condition of data normality was not met, the mean and standard deviation were supplemented with median and lower and upper quartile. The significance level was set to 0.05.

Risk factors were dichotomized or divided into categories for a better interpretation of the results. Age was categorized into years. The length of breastfeeding was dichotomized into 0 = six months or less and 1 = more than six months of breastfeeding, as up to six months, infants are usually exclusively breastfed. The cut-off for the categorization by age at which parents started brushing the infants’ teeth was set to 12 months inclusive. For the frequency of dental hygiene, children were divided into two groups: frequency of teeth brushing less than twice a day, and twice or more times a day.

As BMI is not applicable in children in the same way as in adults, BMI was standardized to a z-score. Z-score of BMI for age and sex was calculated in the Epi Info™ 7.2.4.0 software (Dean et al., 2020); two available growth references were selected: WHO Child Growth Standards (0-5 years) and WHO Reference 2007 (5-19 years). BMI categorization was then performed based on the z-score; children were classified as overweight for z-scores 2 to 3, as “possible risk of overweight” for z-scores 1 to 2, normal weight for z-scores -2 to 1, and underweight for z-scores -3 to -2.

Pearson Chi-Square test was used to evaluate the significance of the difference between children with sECC and the control group in categorical predictors. Alternative Fisher’s exact test was used in the case of low expected counts in groups. T-test and Mann Whitney test were used for continuous and ordinal/non normally distributed data, respectively. The Gamma parameter, a symmetric measure of association, and its p-value were used to express the relationship between the examined risk factors of sECC.

Logistic regression was used to analyze the co-occurrences and interactions of *C. albicans*, *C. dubliniensis*, and *S. mutans*, and differences in their representation between the sECC and control groups, respectively, as well as in groups defined with respect to individual potential risk factors. Where the contingency table contained null values, odds ratio (OR) was calculated in MedCalc (MedCalc Software, Ltd; Odds ratio calculator, ver. 20.009^1^), where 0.5 was added to all values used for the respective OR calculation to be able to assess OR (Pagano and Gauvreau, 2000; Deeks and Higgins, 2010).

Multivariable logistic regression models were constructed for evaluating the complex contribution of investigated potential risk factors for sECC and for maintaining control over observed confounding variables. For this reason, the models included basic descriptive characteristics (sex, age in years, and BMI categories), together with potential microbial and behavioral risk factors.

Models including in addition to the above also analyzed cariogenic and periodontal bacteria were calculated from the subgroup of 157 children in whom the laboratory analysis of these bacteria was performed.

In the multivariable model, the variable selection method was used – specifically, the backward stepwise selection algorithm based on the likelihood-ratio statistics. The algorithm initially used all listed variables and gradually selected the significant ones, i.e., those most contributing to the sECC status of the patient. Nagelkerke R^2^ parameter, a relative measure of the variability explained by a given model, was used solely for model comparison (Nagelkerke, 1991; Mittlböck and Schemper, 1996). The results were supplemented with the value of area under the curve (AUC) from a receiver operating characteristic (ROC) analysis to assess the discrimination ability of the models.

## 3 Results

With the final sample 164 cases and 147 controls, we can detect 12% difference between cases (22%) and controls (10%) in an incidence in a low frequent binary predictor based on the power analysis calculated for 300 individuals with balanced groups (see **Supplementary Material**), 80% power and level of statistical significance 0.05. In higher frequent predictor we can identify 16% difference between cases (66%) and controls (50%). These differences are sufficiently clinically significant, and sample of 311 children was assessed as sufficiently large to achieve the stated aims.

In total, 311 children (169 boys and 142 girls) aged 4.0 ± 0.9 years (mean ± standard deviation, SD) meeting both inclusion and exclusion criteria were included in this study, of which 164 (52.7%) children were diagnosed with sECC (dmft ≥ 6; mean ± SD, 11.5 ± 3.4 dmft, max. 20) and 147 (47.3%) were controls with dmft = 0. Basic characteristics of these two groups of children are shown in **Table 1**. There were no significant differences in gender, age, birth weight, or BMI between the studied groups (p > 0.05); 81.0% of children had normal BMI.

**Table 1:** Basic descriptive characteristics of children classified according to the oral status into cases (with sECC, dmft ≥ 6) and controls (dmft = 0).

Basic characteristics	Descriptive statistics	sECC;n = 164 (52.7%)	Controls;n = 147 (47.3%)	*p*-value†
boys		54.3%	54.4%	0.978
age at the time of examination/sample collection [years]	*mean ± SD*	4.0 ± 1.0	3.9 ± 0.9	0.541
age in categories [years]	< 3	16.5%	17.0%	0.760
	3-4	34.1%	38.1%	
	4-5	31.7%	31.3%	
	5-6	17.7%	13.6%	
birth weight [g]	*mean ± SD*	3 382 ± 473	3 418 ± 416	0.482
BMI z-score^‡^ acc. to WHO	*mean ± SD*	-0.3 ± 1.2	-0.3 ± 1.1	0.857
BMI category acc. to WHO z-score^‡^	underweight	7.9%	8.2%	0.513
	normal weight	79.9%	82.3%	
	possible risk of overweight	8.5%	8.8%	
	overweight	3.7%	0.7%	
breastfeeding		87.8%	92.5%	0.166
breastfeeding at night (n = 145 responses; 43 in sECC group vs. 102 in controls)		88.4%	82.4%	0.365
breastfeeding until the age [months]	*mean ± SD, median (lower; upper quartile)*	11 ± 11, 6 (3; 14)	12 ± 8, 12 (8; 17)	0.001
weaning by or before the 6^th^ month of age		50.6%	23.1%	<0.001
serving sweet beverages		91.5%	64.6%	<0.001
initiation of teeth cleaning [months]	*mean ± SD, median (lower; upper quartile)*	15 ± 7, 12 (12; 24)	11 ± 5, 10 (8; 12)	<0.001
teeth cleaning initiated after the 12^th^ month of age		45.1%	15.0%	<0.001
teeth cleaning frequency [times a day]	*mean ± SD, median (lower; upper quartile)*	1.9 ± 0.6, 2.0 (2.0; 2.0)	1.9 ± 0.4, 2.0 (2.0; 2.0)	0.494
teeth cleaning less than twice a day		22.0%	14.3%	0.081

dmft, number of decay, missing and filled teeth; SD, standard deviation; sECC, severe early childhood caries.

†Statistical significance of the differences between sECC and control groups was tested using the Pearson chi-square test for binary variables, t-test for continuous normally distributed variables (characterized by mean), and Mann-Whitney test for discrete and ordinal variables (characterized using mean and median).

‡BMI z-score is a measure adjusted for sex and age according to WHO child growth standards (The WHO, 2006).

**Figure 1:**
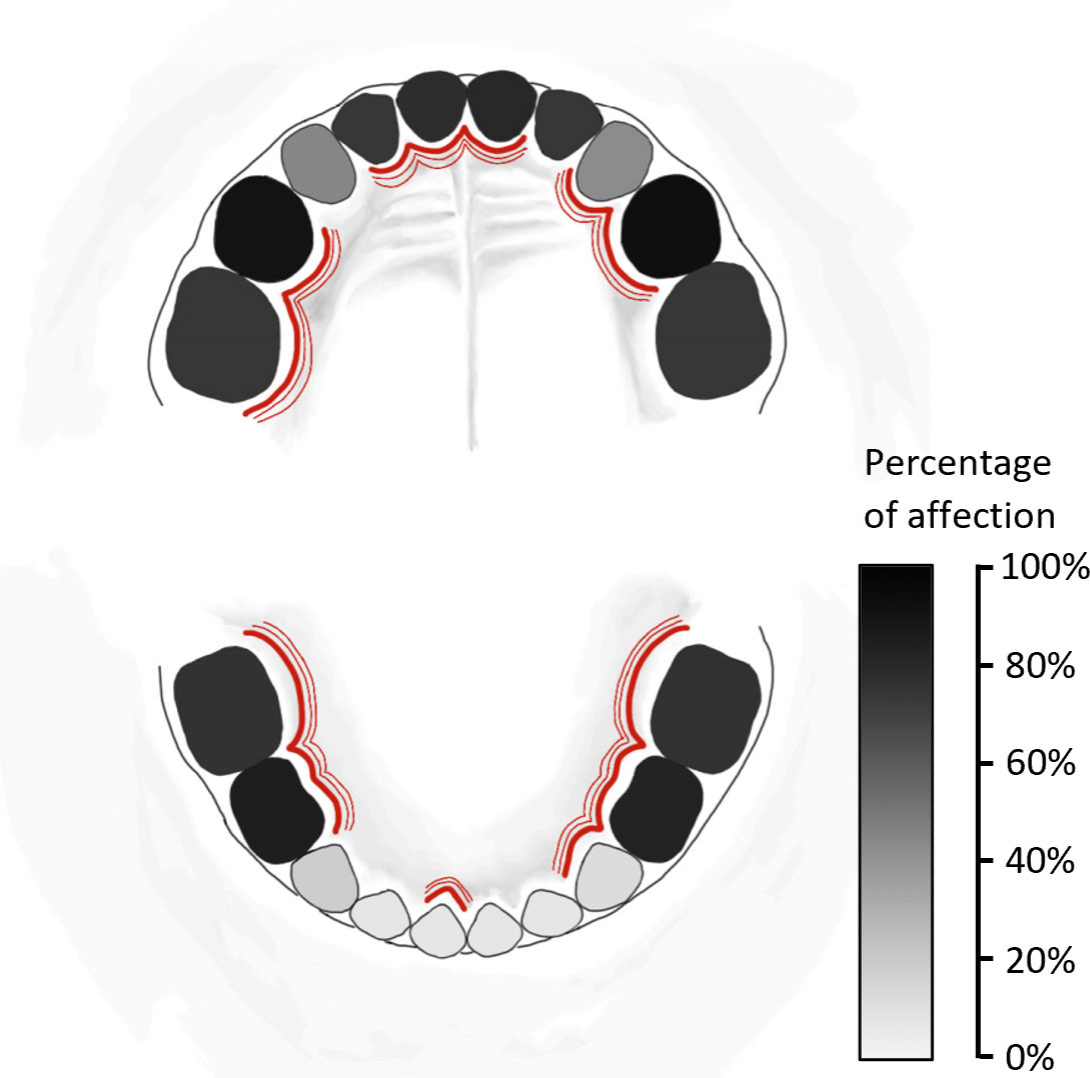
Schema of primary teeth affected by dental caries in all 164 children with severe early childhood caries (sECC, with decay, missing, filled teeth for caries, dmft ≥ 6) included in this study. The percentage of affection of individual teeth by dental caries is expressed using the greyscale. For bacterial sampling, teeth affected by dental caries were selected – these teeth are marked by red lines.

In children with dmft ≥ 6, incisors and canines of the lower jaw (83, 82, 81, 71, 72, 73) were the least affected by dental caries (8.5-17.7%). On the other hand, 45.7% and 43.9% of teeth 53 and 63, respectively, and 77.4-92.7% of other teeth were affected by dental caries in these children (**Figure 1**). Swabs for cariogenic and periodontal bacteria were mostly taken from teeth 54, 51, 64, and 61 (22.6%, 15.1%, 13.2%, and 11.3%, respectively), see **Figure 1**.

### 3.1 Microbial diversity and co-occurrence of *Candida* spp. and *Streptococcus mutans*

**Figure 2:**
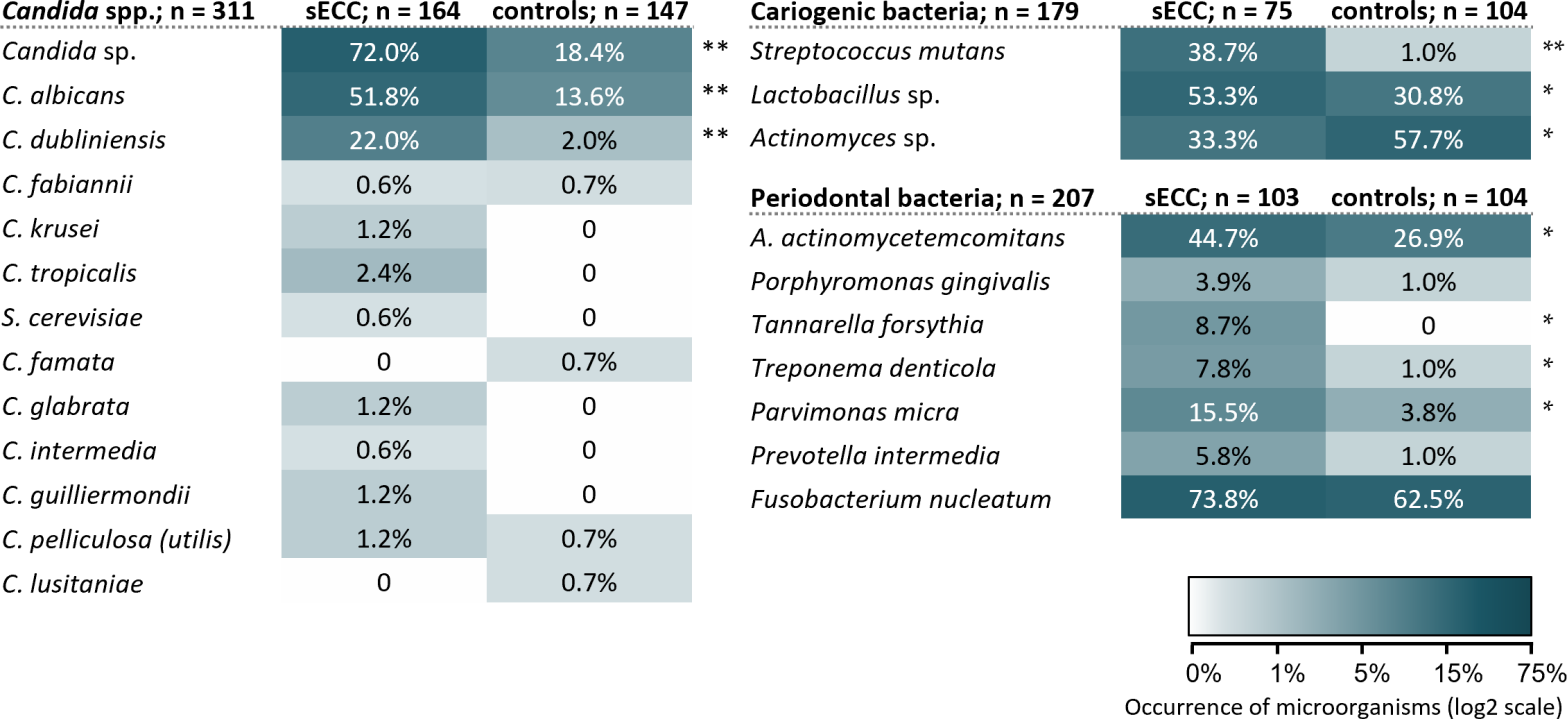
Occurrence and diversity of the investigated oral microorganisms in the subgingival and supragingival dental plaque in the groups of children with sECC and controls. *A. actinomycetemcomitans*, *Aggregatibacter actinomycetemcomitans*; *C.*, *Candida*; *S. cerevisiae*, *Saccharomyces cerevisiae.* */** *p* < 0.05/*p* < 0.001, statistically significant results between group of the children with sECC and controls, the difference is tested with Fisher’s exact test.

In all, 12 *Candida* spp. were identified in our study group (see **Figure 2**). The C*andida* spp. (except for *C. famata* and *C. lusitaniae*), selected cariogenic bacteria (except for *Actinomyces* sp.) and periodontal bacteria were found more frequently in the sECC group than in the control group (see **Figure 2**). The occurrence of *Candida* sp. was significantly associated with sECC with OR (95% CI) of 11.40 (6.65; 19.54), *p* < 0.001. The strongest association was with *C. albicans*, OR 6.83 (3.89; 11.99), *p* < 0.001; *C. dubliniensis*, OR 13.50 (4.06; 44.89), *p* < 0.001; of bacteria, the strongest association was with *S. mutans*, OR 64.93 (8.58; 491.24), *p* < 0.001 (**Figure 2**).

**Figure 3:**
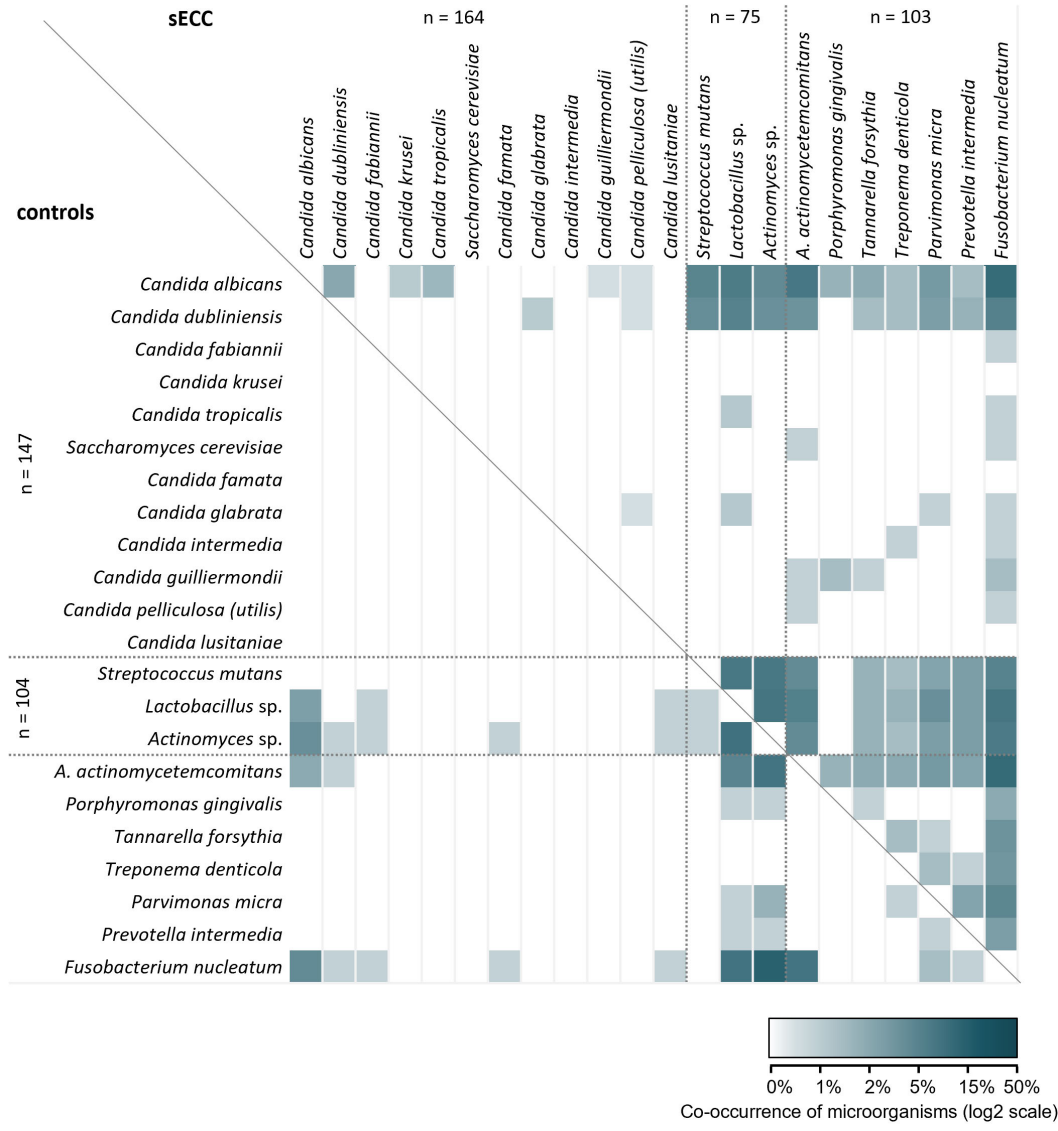
Microbial co-occurrence of *Candida* spp., selected cariogenic and periodontal bacteria in the groups of children with severe early childhood caries (sECC) and controls. Combinations for children with sECC are shown above the diagonal line, those for controls are below the line. *A. actinomycetemcomitans*, *Aggregatibacter actinomycetemcomitans*.

The co-occurrence of individual *Candida* spp. and selected cariogenic and periodontal bacteria is presented in **Figure 3**. It is obvious that in children with sECC, the observed microorganisms occur in combination with other studied species more commonly than in controls. In the control group, no co-occurrence of any *Candida* spp. was observed while in children with sECC, *C. albicans* and *C. dubliniensis* co-occurred together with other *Candida* spp. and cariogenic and periodontal bacteria. Similarly, in the sECC group, we observed combinations of cariogenic and periodontal bacteria more frequently than in controls.

In 26.0% of children in the control group, none of the studied microorganisms were present, which significantly differed from the sECC group (3.8% of children; *p* < 0.001, Mann-Whitney test; data not shown). On average, 2 species of *Candida* or analyzed bacteria were found in the control group compared to 3.6 species in the sECC group. The median (lower, upper quartile) number of studied microorganisms was 2 (0; 3) in the control group and 3 (2; 5) in the sECC group. In 7.6% of children with sECC, as many as 8 or more co-occurring microorganisms were detected while in the control group, no more than 5 microorganisms (in 2.9% of children) were present in the same child.

A closer look at the co-occurrence of the three most significant microorganisms *C. albicans*, *C. dubliniensis*, and *S. mutans* (**Table 2**) revealed that in 4.0% of children with sECC, *C. albicans* and *C. dubliniensis* were identified together (*p* = 0.017, in comparison with 0% in controls). *C. albicans* and *S. mutans* co-existed in 14.7% of children with sECC vs. 0% controls (*p* < 0.001) and almost the same can be said about *C. dubliniensis* and *S. mutans*, the coexistence of which was detected in 10.7% of children with sECC vs. 0% of controls (*p* = 0.002), see **Table 2**. No children with a combination of all three microorganisms were found in our study group. 20.0% of children with sECC and 83.7% of controls were negative for all three species.

**Table 2:** Co-occurrence of *Candida albicans, Candida dubliniensis* and *Streptococcus mutans* relative to sECC.

*Candidaalbicans*	*Candida dubliniensis*	*Streptococcus mutans*	sECC;n = 75 (41.9%)	Controls;n = 104 (58.1%)	OR (95% CI)	p-value
negative	negative	negative	20.0%	83.7%	reference category	
positive	negative	negative	24.0%	13.5%	7.46 (3.07; 18.12)	<0.001
negative	positive	negative	13.3%	1.9%	29.00 (5.77; 145.67)	<0.001
negative	negative	positive	13.3%	1.0%	58.00 (6.91; 486.80)	<0.001
positive	positive	negative	4.0%	0%	39.52 (1.94; 803.34) †	0.017
positive	negative	positive	14.7%	0%	129.84 (7.27; 2 318.85) †	<0.001
negative	positive	positive	10.7%	0%	95.97 (5.27; 1 749.22) †	0.002
positive	positive	positive	0%	0%	―	―

CI, confidence interval; dmft, number of decayed, missing and filled teeth; OR, odds ratio; sECC, severe early childhood caries (dmft ≥ 6); controls, dmft = 0.

†Where null values were present in the contingency table, ORs were calculated in MedCalc.

### 3.2 Behavioral factors

A vast majority of children were breastfed (90.0%), of which breastfeeding at night was reported in 84.1% of children; this was similar in both research groups (*p* > 0.05). However, the proportion of children breastfed for less than 6 months in sECC group was significantly higher than in the control group (50.6% vs. 23.1%, *p* < 0.001). The same was found for children breastfed for up to 12 months (73.2% in cases vs. 55.8% in controls, *p* = 0.001). The analysis of the questionnaires also revealed that 35.4% of children from the control group were not regularly served sweet beverages while in the group with sECC, this was true only for 8.5% of children (*p* < 0.001, **Table 1**). Tooth brushing was initiated after the 1^st^ year of age more frequently in the sECC group than in the group with intact dentition (45.1% vs. 15.0%; *p* < 0.001). 81.7% of children in the entire group brush their teeth at least twice a day, no difference in the frequency of teeth brushing was found between the groups (*p* > 0.05), see **Table 1**.

**Figure 4:**
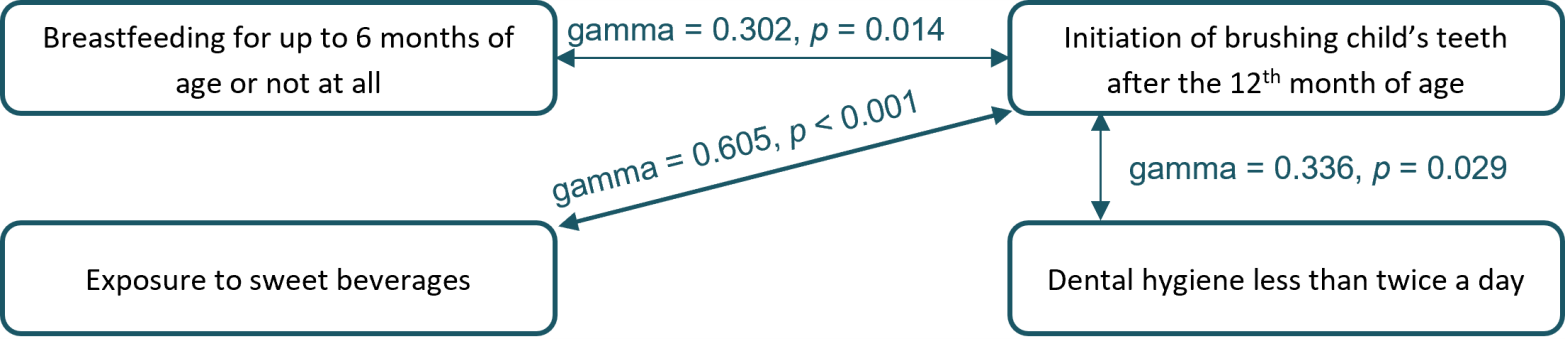
Significant relationships between behavioral risk factors for severe early childhood caries (sECC) development.

The later initiation of brushing children’s’ teeth (after the 12^th^ month of age) was associated with shorter breastfeeding (up to 6 months of age or less), exposure to sweet beverages, and lower frequency of dental hygiene, *p* < 0.05 see **Figure 4**. Of children whose parents started brushing their teeth only after 12 months of age, 47.9% were breastfed for 6 months or less, 91.7% of children were exposed to sweet beverages at the time of the inclusion in the study and 26.0% brushed their teeth less than twice a day. On the other hand, in children whose parents started brushing their teeth by or before the 12^th^ month of age, 33.0% of children were breastfed for 6 months or less, 73% regularly drink sweet beverages and 14.9% brush their teeth less than twice a day.

### 3.3 Microbial and behavioral risk factors for sECC

Relations between the presence of *Candida* sp., *C. albicans*, *C. dubliniensis* and *S. mutans*, and selected behavioral factors (the length of breastfeeding, regular consumption of sweet beverages, age at the beginning of teeth brushing, and the frequency of teeth brushing) are described in **Supplementary Table S1**. *C. dubliniensis* and *S. mutans* were significantly more common in the dental plaque of children who were breastfed for less than 6 months (*p* < 0.05). Similarly, serving sweet beverages was associated with a higher frequency of the occurrence of *Candida* spp. and *S. mutans* (*p* < 0.05, OR > 3, **Supplementary Table S1**). Initiation of regular teeth brushing after the 12^th^ month of child’s age was associated with a more common occurrence of these candidas (*p* < 0.05), *C. albicans* was associated also with a lower frequency of teeth brushing (*p* < 0.05, **Supplementary Table S1**).

As the only bacterium, *Actinomyces* sp. occurred less frequently in children whose parents started brushing their teeth only after the 12^th^ month of age (22.0%) than in children whose parents started brushing their teeth earlier (55.1%); OR (95% CI): 0.23 (0.10; 0.52), *p* < 0.001 (data not shown).

### 3.4 Multivariable logistic regression model of risk factors for sECC

We have built a multivariable model including the occurrence of *C. albicans* and *C. dubliniensis*, and four selected behavioral factors (breastfeeding for 6 months or less, exposure to sweet beverages, beginning of brushing child´s teeth after the 12^th^ month of age, and dental hygiene less than twice a day) to estimate their effects on sECC occurrence (see **Table 3**). **Table 3** presents individual contributions and statistical significance of selected individual parameters in relation to sECC according to the results of the univariate model.

**Table 3:** Association between potential microbial and behavioral risk factors for sECC in univariate and multivariable model based on the entire study group (n = 311).

	sECC vs. controls (univariate model)		sECC vs. controls(multivariable model†)	
Descriptive characteristics and potential risk factors used as predictors	OR (95% CI)	*p*-value	OR_multi_ (95% CI)	*p*-value_multi_
**Descriptive parameters**				
female	1.01 (0.64; 1.57)	0.978	–	
age in years	1.11 (0.88; 1.40)	0.398	–	
BMI in categories according to z-score	1.26 (0.81; 1.98)	0.306	–	
***Candida* spp.**				
*Candida albicans*	6.83 (3.89; 11.99)	< 0.001	9.62 (4.99; 18.54)	< 0.001
*Candida dubliniensis*	13.50 (4.06; 44.89)	< 0.001	16.93 (4.70; 60.99)	< 0.001
**Behavioral factors**				
breastfeeding for 6 months or less	3.41 (2.09; 5.56)	< 0.001	2.71 (1.45; 5.07)	0.002
exposure to sweet beverages	5.87 (3.08; 11.16)	< 0.001	3.77 (1.73; 8.19)	0.001
beginning of brushing child’s teeth after the 12^th^ month of age	4.67 (2.70; 8.08)	< 0.001	4.10 (2.11; 7.98)	< 0.001
dental hygiene less than 2times per day	1.69 (0.93; 3.05)	0.083	–	

CI, confidence interval; dmft, number of decayed, missing and filled teeth; OR, odds ratio; sECC, severe early childhood caries (dmft ≥ 6); controls, dmft = 0; multi, OR, CI and p-values from the multivariable model.

†All variables detailed in the table were used for the construction of the multivariable model calculated using logistic regression with the backward stepwise selection algorithm based on the “likelihood ratio” statistics.

The multivariable model (see Methods for a detailed description) includes multiplicative contribution of predictors, see the results of the multivariable model in **Table 3**. Our data imply that the presence of *C. albicans* (OR 9.62) and *C. dubliniensis* (OR 16.93) is significantly associated with the increased risk of sECC. This contribution is independent of other risk factors and even higher after adjusting for the other risk factors (ORs increased in the multivariable model in comparison with the univariate model). Of behavioral factors, the length of breastfeeding is also significantly associated with the development of sECC status. The absence of breastfeeding or weaning within the first 6 months of age increases the odds of sECC 2.71 times compared to the longer period of breastfeeding adjusted for other predictors (see **Table 3**). Children exposed to sweet beverages have 3.77 times higher odds of sECC than children who do not drink sweet drinks. The age at the beginning of teeth brushing might be another parameter in sECC prediction since the beginning after the 12^th^ month of age was associated with 4.10 times higher odds of sECC. ORs of all behavioral risk factors were reduced in the multivariable model compared with the univariate models.

The above-described resulting model explains 51.1% of the variability in the case-control definition in our data (according to Nagelkerke R^2^ parameter) and well discriminates cases and controls in our dataset, correctly classifying 87.1% (*p* < 0.001) according to the ROC analysis, see **Table 4**. If the model description of sECC is calculated using the same parameters but only *C. albicans* is included, the model explains 42.9% of the variability and after excluding the *Candida* spp. at all, the resulting model explains only 29.8% of the variability. In other words, adding *C. dubliniensis* into the model leads to explaining 8.2% more variability than without it, and the model with both yeasts explains 21.1% more variability than the model containing only behavioral risk factors, see **Table 4**. A model including only candidas (and descriptive parameters that are included in all models) interprets 37.4% of variability, which means that the presence of *Candida* spp. is a better descriptor of sECC than the behavioral factors alone (29.8%).

**Table 4:** Comparison of multivariable models predicting sECC status.

Multivariable models n = 164 cases with sECC and 147 controls	Nagelkerke R^2^ parameter	AUC (95% CI) †
model in **Table 3**	0.511	0.871 (0.832; 0.909)
model from the same parameters as in **Table 3**, **without** *C. dubliniensis*	0.429	0.827 (0.782; 0.872)
model from the same parameters as in **Table 3**, **without** *C. albicans* and *C. dubliniensis*	0.298	0.758 (0.706; 0.811)
model from the same parameters as in **Table 3**, **without** behavioral factors	0.374	0.780 (0.728; 0.832)
**Multivariable models on a subgroup of patients** n = 53 cases and 104 controls		
model from the same parameters as in **Table 3**, + ***Streptococcus mutans***	0.644	0.915 (0.865; 0.964)
model from the same parameters as in **Table 3**, + ***Streptococcus mutans* without** *C. albicans* and *C. dubliniensis*	0.520	0.872 (0.812; 0.932)
**full model** =model from the same parameters as in **Table 3**, + cariogenic and periodontal bacteria	0.751	0.948 (0.909; 0.987)
full model **without** *Streptococcus mutans*	0.647	0.923 (0.879; 0.967)
full model **without** *C. albicans* and *C. dubliniensis*	0.613	0.909 (0.856; 0.963)
full model **without** *C. albicans*, *C. dubliniensis* and behavioral factors	0.577	0.885 (0.821; 0.948)

AUC, area under the curve from ROC analysis, describes discrimination ability of the model, AUC > 0.9 suggests an excellent model, AUC = 0.5 suggests useless model; CI, confidence interval; dmft, number of decayed, missing and filled teeth; sECC, severe early childhood caries (dmft ≥ 6); controls (dmft = 0).

†All AUC statistically significant, p < 0.001.

Moreover, we have also constructed models based on the data from the subgroup of patients (see **Supplementary Figure S1**) in whom the selected bacteria were analyzed. In these models, cariogenic and periodontal bacteria were included in addition to the factors used for the entire patient group. The results of these models are shown in **Table 4**. Inclusion of the bacteria into the full model led to an improvement of explained variability to 75.1% and the correct classification of 94.8% of patients.

## 4 Discussion

The human oral cavity contains more than 700 bacterial species; the approximate number of *Candida* sp. in that localization remains, however, unclear (Montelongo-Jauregui and Lopez-Ribot, 2018). This is largely caused by the fact that *Candida* sp. were ignored for a long time and the oral microbiome research focused predominantly on bacteria. Lately, however, the interest in the identification of the role of candidas in health and diseases of the oral microbiome grows (Diaz et al., 2014). From the clinical point of view, a coinfection with *C. albicans* and *S. mutans* is strongly associated with the relapse of dental caries (Lemos et al., 2013; Metwalli et al., 2013).

### 4.1 The association of analyzed microorganisms with sECC

In our study, a higher occurrence of *Candida* spp., especially *C. albicans* and *C. dubliniensis*, (see **Figure 2**) and of most analyzed cariogenic and periodontal bacteria was detected in children with sECC compared to controls. The diversity of all analyzed microorganisms was also higher in the dental plaque of children with sECC. This is in accordance with the results of the study on the Canadian First Nation population (Agnello et al., 2017), in which the dental plaque of children with sECC contained significantly higher levels of known cariogenic microorganisms than plaque from their caries-free counterparts. In line with our findings, O’Connell et al. (2020) documented a higher beta diversity in children with active caries in comparison with caries-free children. *C. albicans* and *C. dubliniensis* were both previously associated with sECC (Al-Ahmad et al., 2016; O’Connell et al., 2020). de Jesus et al. (2020) reported that the occurrence of *C. albicans* did not differ between children without caries and those with sECC, while the representation of *C. dubliniensis* was significantly lower in caries-free children. However, unlike the presented study, none of these studies evaluated a complex interplay between the microorganisms and behavioral factors.

The co-occurrence of *Candida* spp. with bacteria was more common in children with sECC than in controls (see **Figure 3**). More specifically, *C. albicans* and *C. dubliniensis* co-occurred with *S. mutans* only in sECC children (see **Table 2**). This indicates that an interaction between these candidas and bacteria may create conditions leading to sECC development. It was previously suggested that *C. albicans* as well as *C. dubliniensis* “participate in the disease process by synergistically interacting with acidogenic bacteria” (Diaz and Dongari-Bagtzoglou, 2021). We assume, same as Kirkpatrick et al. (2000), that *C. albicans* and *C. dubliniensis* compete for resources, as their co-occurrence was relatively rare; mostly, just one of these candidas was present (see **Table 2**). The co-occurrence of *C. albicans* or *C. dubliniensis* together with *S. mutans* greatly increased the odds of sECC (see **Table 2**). This is in accordance with previous studies focusing on *C. albicans* and *S. mutans*; however, these studies did not investigate the role of *C. dubliniensis* (Xiao et al., 2018a, Garcia et al., 2021).

### 4.2 Behavioral risk factors in sECC children

In accordance with Kraljevic et al. (2017), we found the beginning of brushing child’s teeth after the 12^th^ month of age to be the strongest behavioral risk factor. A later initiation of brushing a child’s teeth correlated with the exposure to sweet beverages and mildly with the shorter time of breastfeeding (see **Figure 4**), another risk factors. The fact that breastfeeding lasting for more than six months seems to be a protective factor in our study, might be caused by the fact that mothers who care more about a healthy lifestyle are more interested in childcare and upbringing and do breastfeed their child longer (which agrees with the general WHO recommendations). They are also more likely to monitor the intake of sugars, as well as sweet beverages, as they are aware of the negative effects of the presence of unhealthy sugars in the diet and are probably more educated in the importance of early dental care. Hong et al. (2014) found that mothers who breastfed for ≥ 6 months had higher educational status and associated longer breastfeeding with lower mean values of decayed and filled surfaces.

In some works (Mohebbi et al., 2008; Feldens et al., 2010; van Meijeren-van Lunteren et al., 2021), they observed the opposite effect than we and long breastfeeding (over 12 months) was reported to be a risk factor of ECC while in others (Kraljevic et al., 2017), this was not observed. This finding may be related to the socio-economic status of the study population; for example, in developing countries, children are much longer breastfed due to the absence of safe water or lack of food, so long breastfeeding alone might not be the real protective (or risk) factor influencing sECC development in such countries. In Feldens et al. (2010), prolonged breastfeeding was risky for sECC and most of these mothers had ≤ 8 years of schooling. Thus, the length of breastfeeding must be interpreted with caution and differently in high- and low-income/education populations as it seems to be rather a confounding variable with a potential to represent various behaviors depending on the community studied.

### 4.3 Microbial and behavioral risk factors for sECC in a model

We have built a multivariable model, which showed the higher odds of sECC occurrence in children, in whom selected microbial and behavioral risk factors were observed. The investigated behavioral risk factors turned out to be less important in the multivariable model than when their individual associations with sECC occurrence were analyzed (which is demonstrated by the reduction of their ORs in the multivariable model in comparison with the univariate one, see **Table 3**). On the contrary, the importance of the presence of *C. albicans* and *C. dubliniensis* was higher when considered together with behavioral risk factors (i.e., their ORs increased in the multivariable model, see **Table 3**).

The model including *S. mutans* as the only microbial representative explained a similar amount of variability (52%) as the model with *C. albicans* and *C. dubliniensis* only (51.1%). When these three microorganisms were considered together, the explained variability increased to 64.6%. This implies, that besides *S. mutans*, *C. albicans* and *C. dubliniensis* should be also watched for. It is necessary to mention that Cariogram, a software presently available for dental caries risk assessment (Bratthall and Hänsel Petersson, 2005), uses the occurrence of *S. mutans* as the only microbial risk factor for its predictions. In view of the above, we hypothesized that if the association between *Candida* spp. and sECC is confirmed, their presence could constitute a valuable addition to the model for risk assessment of sECC.

The full model including bacteria as well as yeasts built on a subgroup of patients and controls showed an even better ability to interpret the variability in cases and controls (75.1%) in comparison with the model containing only *C. albicans* and *C. dubliniensis* (explaining 51.1% of the variability, see **Table 4**). On the other hand, the model including only bacteria was able to interpret 61.3% of the variability. This is 10.2% more than the model containing only candidas, but still 14.2% less than the “full” model including both candidas and bacteria, which, again, confirms that it would be beneficial to include all three microorganisms in the evaluation of the risk for ECC.

### 4.4 Strength, shortcomings and limitations of our study

The strength of our case-control study lies in the design, where two extreme phenotypes were compared. This approach is desirable in studies of multifactorial diseases (Setia, 2016). Moreover, strict inclusion and exclusion criteria were applied to eliminate bias. Exclusion of children with low birth weight prevented the presence of dental caries due to possible enamel defects associated with premature birth (Cruvinel et al., 2012; Corrêa-Faria et al., 2013; Schüler et al., 2018). For the same reason, we also excluded children with severe diseases, which can also affect their immunity and, therefore, the environment of the oral cavity. We also excluded severely under- and overweight children as these children might be nutritionally deficient and their immune system negatively affected (Janakiram et al., 2018).

In addition, the size of the groups of cases and controls was comparable, and the groups were balanced in terms of ethnicity (Czech Caucasian), sex, age, birth weight, and BMI-for-age (**Table 1**). The number of participants in our study was unusually large (164 cases and 147 controls) compared to other studies dealing with *Candida* sp. and ECC containing typically a total number of around 50 children with primary dentition (Al-Ahmad et al., 2016; Agnello et al., 2017; de Jesus et al., 2020; O’Connell et al., 2020). Another benefit of our work lies in the method used for the identification of *Candida* spp. The MALDI-TOF MS method reliably distinguished *C. dubliniensis* from *C. albicans.* Such identification was not performed in previous studies (Baillie and Douglas, 1999; Jabra-Rizk et al., 2006; Ayadi et al., 2020), which led to the overestimation of the association of *C. albicans* with sECC.

Our study comes with some limitations as well. Firstly, it would be certainly beneficial to have data on colonization by cariogenic bacterial from all samples as it would support the robustness of modelling. However, at the beginning of this long-term project, the methods facilitating easy analysis of these bacteria were not available; this only changed later during the study period. However, we still believe that even a subgroup of 153 patients is large enough for creating a model with sufficient reliability.

In this study, the socio-economic status of the participants was not evaluated as this information was not available for all subjects. In the subgroup in whom these data were recorded (n = 134 children), mothers of caries-free children had more often university education (74%), than mothers of children with sECC (23%). However, these data must be interpreted with caution because they are burdened with a volunteer effect when mothers of children with intact dentition were more likely to both enrol their healthy children in the study and, at the same time, these mothers were more likely to declare their university education than educated mothers whose children suffered from sECC. Based on the results from other studies (Feldens et al., 2010; Hong et al., 2014; Agnello et al., 2017), we can expect that in our country, higher education leads to prolonged breastfeeding (seen as protective in our study) and earlier start of children’s teeth brushing.

In addition, teeth brushing with fluoride toothpaste and using fluoridated water (Toumba et al., 2019) were not considered in this study; water fluoridation is not available in the Czech Republic since 1988 so this factor does not play a role in our analysis, being equally valid for both groups; the use of fluoridated toothpaste was not evaluated, either, because the brands and types of toothpaste used in the families are typically changing over time so it was impossible to track this parameter objectively.

### 4.5 Future direction in studies focusing on sECC risk assessment and conclusions

In this study, we investigated a wide spectrum of microbial and behavioral parameters possibly involved in the onset and progression of dental caries in the primary dentition. Results imply that our hypothesis was correct and *Candida* sp. (as a whole group or, even better, specifically distinguishing *C. albicans* and *C. dubliniensis*), might constitute a valuable addition for the algorithms predicting the risk of development of sECC. We suggested *C. albicans* and *C. dubliniensis* to be one of the “keystone pathogens” for sECC. Future prospective studies on sECC should include *Candida* spp. and distinguish between *C. albicans* and *C. dubliniensis* as, according to our data, children with sECC were more likely to be infected by *C. dubliniensis* even more than by *C. albicans* compared to caries-free children. However, it is essential to include also behavioral factors and bacteria associated with dental caries in children. A complex model estimating the risk of sECC development should ideally include host genetic background as well.

## Data availability statement

The raw data supporting the conclusions of this article will be made available by the corresponding authors, without undue reservation.

## Ethics statement

The studies involving human participants were reviewed and approved by Institutional Review Board (or Ethics Committee) of Masaryk University (protocol code 3/2004, 3rd March, 2004) and the St. Anne´s University Hospital in Brno, Czech Republic (protocol code 1G/2017, 24th June, 2016). Written informed consent to participate in this study was provided by the participants’ legal guardian/next of kin.

## Author contributions

MC, BL, and PBL drafted the paper, designed and created figures. ZD, DG, LD, JJ, FR, VH, MK, LIH, and MB critically revised the manuscript. PBL, MC, and ZD designed this study. MC, PBL, ZD, BL, LD, and JJ provided analysis, or interpretation of data for the work. FR and VH were responsible for the design of method and *Candida* spp. analysis. PBL and DG were responsible for sample storing, design of microbial analysis and/or its providing and database management. MK, LIH, and MB were responsible for the clinical part of study and samples collection. FR and MK initiated the study. PBL and LIH secured funding for the study. All authors revised the final version of the manuscript.

## Funding

This research was supported by Ministry of Health of the Czech Republic, grant number: IGA NR8394-3/2005, NV17-30439A, NU20-08-00205, and by a project provided by University Hospital Brno, Ministry of Health Czech Republic – RVO (FNBr, 65269705). This publication has received funding from the European Union’s Horizon 2020 Research and Innovation Programme under grant agreement No 857560. This publication reflects only the author’s view and the European Commission is not responsible for any use that may be made of the information it contains. Authors also thank the Research Infrastructure RECETOX RI (No LM2018121) and project CETOCOEN EXCELLENCE (No CZ.02.1.01/0.0/0.0/17_043/0009632) financed by the Ministry of Education, Youth and Sports for supportive background.

## Acknowledgments

We would like to thank our colleagues, who helped us with samples collection: Kristina Musilova, Lenka Zackova, Dominika Hulova, Margarita Rousi, Zuzana Zidekova, Denisa Kavrikova, and Zdenek Linhart. We also thank Jaroslav Janosek and Albert Ksinan for critical review of the manuscript, and Jan Lochman for management of the multiplex qPCR analysis.

## Conflict of interest

The authors declare that the research was conducted in the absence of any commercial or financial relationships that could be construed as a potential conflict of interest.

## Publisher’s note

All claims expressed in this article are solely those of the authors and do not necessarily represent those of their affiliated organizations, or those of the publisher, the editors and the reviewers. Any product that may be evaluated in this article, or claim that may be made by its manufacturer, is not guaranteed or endorsed by the publisher.

## Footnotes

1 Available at www.medcalc.org/calc/odds_ratio.php

## Supplementary material

Supplementary Figure 1 Overall flowchart describing recruitment of participants for this study.

Supplementary Table 1 Occurrences of *Candida* sp., *Candida albicans*, *Candida dubliniensis*, and *Streptococcus mutans* in association with risk factors for sECC.
