# Supplementary material for: Candida species and selected behavioral factors co-associated with severe early childhood caries: Case-control study
Source: Front Cell Infect Microbiol. 2022 Jul 25;12:943480. doi: 10.3389/fcimb.2022.943480 (PMC9357982; doi:10.3389/fcimb.2022.943480)
Supplement: Supplementary Figure 1 — Overall flowchart describing recruitment of participants for this study. [file DataSheet_1.docx]

Supplementary Material

**Power analysis**

The power analysis was computed for the identification of detectable difference in binary endpoint occurrence between groups of patients under the following settings:

- Power 0.8, level of statistical significance 0.05
- Total sample size 300
- Proportion of endpoint occurrence in reference group 10%, 25%, 50%

| **N in group of cases** | **N in group of controls** | **N total** | **Probability of endpoint occurrence – cases** | **Probability of endpoint occurrence – controls** | **Difference** |
| --- | --- | --- | --- | --- | --- |
| 150 | 150 | 300 | 22% | 10% | 12% |
| 150 | 150 | 300 | 40% | 25% | 15% |
| 150 | 150 | 300 | 66% | 50% | 16% |

Analysis was computed using PASS 16 Power Analysis and Sample Size Software (2018). NCSS, LLC. Kaysville, Utah, USA, ncss.com/software/pass.

**References**

Chow, S.C., Shao, J., and Wang, H. 2008. Sample Size Calculations in Clinical Research, Second Edition. Chapman & Hall/CRC. Boca Raton, Florida.

D'Agostino, R.B., Chase, W., and Belanger, A. 1988. 'The Appropriateness of Some Common Procedures for Testing the Equality of Two Independent Binomial Populations', The American Statistician, August 1988, Volume 42 Number 3, pages 198-202.

Fleiss, J. L., Levin, B., and Paik, M.C. 2003. Statistical Methods for Rates and Proportions. Third Edition. John Wiley & Sons. New York.

Lachin, John M. 2000. Biostatistical Methods. John Wiley & Sons. New York.

Machin, D., Campbell, M., Fayers, P., and Pinol, A. 1997. Sample Size Tables for Clinical Studies, 2nd Edition. Blackwell Science. Malden, Mass.

Ryan, Thomas P. 2013. Sample Size Determination and Power. John Wiley & Sons. Hoboken, New Jersey.

**
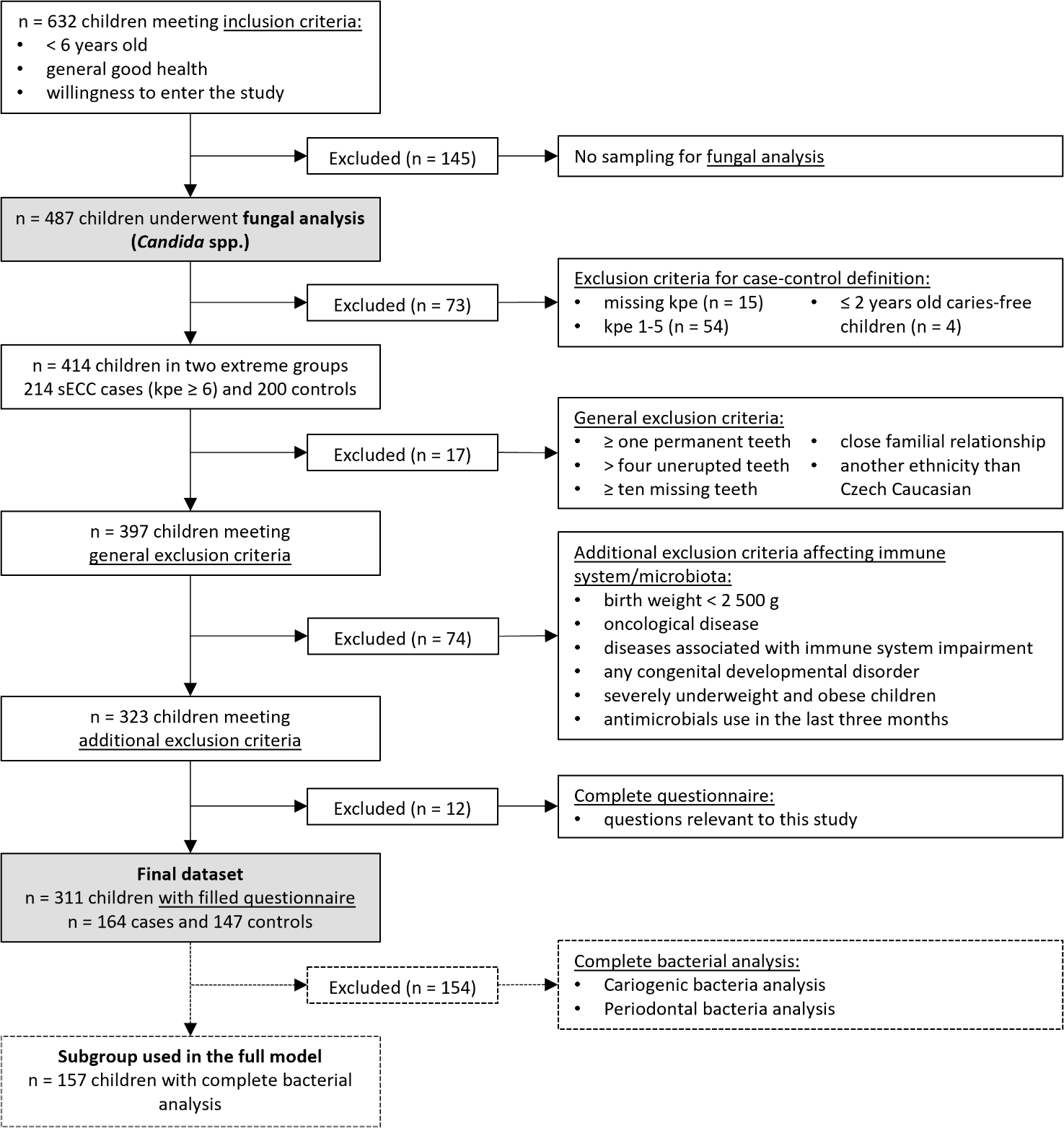
**

**Supplementary Figure S1.** Overall flowchart describing recruitment of participants for this study

**Supplementary Table S1.** Occurrences of *Candida* sp., *Candida albicans*, *Candida dubliniensis,* and *Streptococcus mutans* in association with risk factors for sECC

| **n = 311 †; row %** | ***Candida* sp.** | ***C. albicans*** | ***C. dubliniensis*** | ***S. mutans*; n = 179 †** |
| --- | --- | --- | --- | --- |
| **Length of breastfeeding** |  |  |  |  |
| 6 months or less (37.6%) | 58.1% | 38.5% | 23.1% | 25.0% |
| more than 6 months (62.4%) | 39.7% | 30.9% | 6.2% | 11.7% |
| OR (95% CI) | 2.11 (1.32; 3.36) | 1.40 (0.86; 2.26) | 4.55 (2.20; 9.40) | 2.51 (1.13; 5.58) |
| *p*-value | 0.002 | 0.174 | <0.001 | 0.024 |
| **Consumption of sweet beverages** |  |  |  |  |
| yes (78.8%) | 52.2% | 38.4% | 14.7% | 20.5% |
| no (21.2%) | 25.8% | 16.7% | 4.5% | 7.7% |
| OR (95% CI) | 3.15 (1.72; 5.78) | 3.11 (1.55; 6.25) | 3.62 (1.08; 12.14) | 3.09 (1.02; 9.35) |
| *p*-value | <0.001 | 0.001 | 0.037 | 0.046 |
| **Beginning of teeth brushing** |  |  |  |  |
| after the 12^th^ month of age (30.9%) | 57.3% | 42.7% | 18.8% | 22.0% |
| by or before the 12^th^ month of age (69.1%) | 41.9% | 29.8% | 9.8% | 15.2% |
| OR (95% CI) | 1.86 (1.15; 3.03) | 1.76 (1.07; 2.90) | 2.13 (1.08; 4.22) | 1.57 (0.65; 3.75) |
| *p*-value | 0.012 | 0.027 | 0.030 | 0.313 |
| **Frequency of teeth brushing** |  |  |  |  |
| less than twice a day (18.3%) | 59.6% | 47.4% | 19.3% | 11.1% |
| twice a day and more frequently (81.7%) | 43.7% | 30.7% | 11.0% | 17.8% |
| OR (95% CI) | 1.90 (1.06; 3.42) | 2.03 (1.13; 3.64) | 1.93 (0.90; 4.15) | 0.58 (0.16; 2.06) |
| *p*-value | 0.031 | 0.017 | 0.093 | 0.399 |

sECC, severe early childhood caries

† Bacterial sampling was available in 179 children; in 22.9% of them, teeth brushing began after the 12^th^ month of age; teeth were brushed less than twice a day in 15.1%; 38.0% of children were fed until the 6^th^ month or less and sweet beverages are regularly served to 70.9% of children.
